# Supplementary material for: Modeling glioblastoma heterogeneity as a dynamic network of cell states
Source: Mol Syst Biol. 2021 Sep 16;17(9):e10105. doi: 10.15252/msb.202010105 (PMC8444284; doi:10.15252/msb.202010105)
Supplement: Supplementary file 6 — Source Data for Figure 5 [file MSB-17-e10105-s004.zip › Figure5A_sourcedata/GSEA_3017/nosigdb_stateD.GseaPreranked.1622795010397/DALMO_BARCODENUMBER_UNPUB.html]

Details for gene set DALMO\_BARCODENUMBER\_UNPUB[GSEA]

|  || Dataset | state23017 |
| Phenotype | NoPhenotypeAvailable |
| Upregulated in class | na\_neg |
| GeneSet | DALMO\_BARCODENUMBER\_UNPUB |
| Enrichment Score (ES) | -0.62227845 |
| Normalized Enrichment Score (NES) | -3.3764327 |
| Nominal p-value | 0.0 |
| FDR q-value | 0.0 |
| FWER p-Value | 0.0 |
Table: GSEA Results Summary

  

Fig 1: Enrichment plot: DALMO\_BARCODENUMBER\_UNPUB      
 Profile of the Running ES Score & Positions of GeneSet Members on the Rank Ordered List

  

| PROBE | GENE SYMBOL | GENE\_TITLE | RANK IN GENE LIST | RANK METRIC SCORE | RUNNING ES | CORE ENRICHMENT || 1 | PTTG1 |  |  | 14 | 0.490 | -0.0038 | No |
| 2 | SMS |  |  | 45 | 0.358 | -0.0229 | No |
| 3 | HMGB3 |  |  | 80 | 0.311 | -0.0459 | No |
| 4 | LDHA |  |  | 92 | 0.297 | -0.0503 | No |
| 5 | TROAP |  |  | 109 | 0.283 | -0.0590 | No |
| 6 | LSM5 |  |  | 123 | 0.274 | -0.0654 | No |
| 7 | BIRC5 |  |  | 130 | 0.271 | -0.0661 | No |
| 8 | PHPT1 |  |  | 189 | -0.253 | -0.1098 | No |
| 9 | MKI67 |  |  | 206 | -0.254 | -0.1190 | No |
| 10 | GPC1 |  |  | 213 | -0.255 | -0.1199 | No |
| 11 | ITGAV |  |  | 217 | -0.255 | -0.1184 | No |
| 12 | PRR11 |  |  | 361 | -0.269 | -0.2317 | No |
| 13 | FOXM1 |  |  | 478 | -0.282 | -0.3227 | No |
| 14 | DDR1 |  |  | 491 | -0.284 | -0.3281 | No |
| 15 | NUDT14 |  |  | 514 | -0.287 | -0.3417 | No |
| 16 | LAMB2 |  |  | 558 | -0.292 | -0.3725 | No |
| 17 | PROS1 |  |  | 613 | -0.303 | -0.4121 | No |
| 18 | PLK4 |  |  | 699 | -0.319 | -0.4770 | No |
| 19 | KIF4A |  |  | 706 | -0.320 | -0.4769 | No |
| 20 | IGFBP7 |  |  | 733 | -0.326 | -0.4931 | No |
| 21 | SPC24 |  |  | 747 | -0.330 | -0.4986 | No |
| 22 | GSTM3 |  |  | 763 | -0.335 | -0.5057 | No |
| 23 | IDS |  |  | 782 | -0.339 | -0.5152 | No |
| 24 | CDC20 |  |  | 793 | -0.341 | -0.5180 | No |
| 25 | H2AFX |  |  | 810 | -0.347 | -0.5257 | No |
| 26 | CENPM |  |  | 818 | -0.349 | -0.5260 | No |
| 27 | MAD2L1 |  |  | 819 | -0.350 | -0.5205 | No |
| 28 | RRM2B |  |  | 839 | -0.355 | -0.5306 | No |
| 29 | CHPF |  |  | 863 | -0.360 | -0.5438 | No |
| 30 | TK1 |  |  | 902 | -0.375 | -0.5692 | No |
| 31 | TUBB4B |  |  | 916 | -0.380 | -0.5739 | No |
| 32 | KIF18B |  |  | 935 | -0.388 | -0.5826 | No |
| 33 | DIAPH3 |  |  | 943 | -0.390 | -0.5822 | No |
| 34 | TMEM59L |  |  | 952 | -0.396 | -0.5826 | No |
| 35 | SKA3 |  |  | 965 | -0.399 | -0.5862 | No |
| 36 | FANCD2 |  |  | 976 | -0.405 | -0.5880 | No |
| 37 | SPDL1 |  |  | 995 | -0.412 | -0.5964 | No |
| 38 | KIF11 |  |  | 1003 | -0.416 | -0.5956 | No |
| 39 | PLXNB2 |  |  | 1015 | -0.423 | -0.5980 | No |
| 40 | PCDH9 |  |  | 1019 | -0.427 | -0.5937 | No |
| 41 | NCAPG2 |  |  | 1027 | -0.430 | -0.5927 | No |
| 42 | TPX2 |  |  | 1034 | -0.437 | -0.5908 | No |
| 43 | ZBTB20 |  |  | 1061 | -0.451 | -0.6051 | No |
| 44 | SMC2 |  |  | 1062 | -0.452 | -0.5980 | No |
| 45 | PLTP |  |  | 1068 | -0.454 | -0.5950 | No |
| 46 | PLK1 |  |  | 1082 | -0.460 | -0.5984 | No |
| 47 | RACGAP1 |  |  | 1112 | -0.479 | -0.6148 | Yes |
| 48 | LONRF2 |  |  | 1114 | -0.479 | -0.6080 | Yes |
| 49 | SEZ6L2 |  |  | 1126 | -0.492 | -0.6094 | Yes |
| 50 | MDM2 |  |  | 1135 | -0.499 | -0.6081 | Yes |
| 51 | PHLDA3 |  |  | 1137 | -0.501 | -0.6010 | Yes |
| 52 | IFI27L2 |  |  | 1145 | -0.506 | -0.5988 | Yes |
| 53 | CKAP2L |  |  | 1147 | -0.508 | -0.5917 | Yes |
| 54 | NUSAP1 |  |  | 1154 | -0.515 | -0.5885 | Yes |
| 55 | MIS18BP1 |  |  | 1162 | -0.524 | -0.5860 | Yes |
| 56 | GRIA2 |  |  | 1164 | -0.526 | -0.5786 | Yes |
| 57 | CIP2A |  |  | 1165 | -0.533 | -0.5702 | Yes |
| 58 | SGO2 |  |  | 1176 | -0.546 | -0.5699 | Yes |
| 59 | KIF2C |  |  | 1183 | -0.558 | -0.5660 | Yes |
| 60 | DKK3 |  |  | 1184 | -0.559 | -0.5572 | Yes |
| 61 | CDCA3 |  |  | 1187 | -0.563 | -0.5500 | Yes |
| 62 | ASPM |  |  | 1189 | -0.567 | -0.5419 | Yes |
| 63 | NUF2 |  |  | 1194 | -0.574 | -0.5362 | Yes |
| 64 | DHFR |  |  | 1197 | -0.576 | -0.5288 | Yes |
| 65 | CKS1B |  |  | 1198 | -0.577 | -0.5197 | Yes |
| 66 | GTSE1 |  |  | 1210 | -0.602 | -0.5193 | Yes |
| 67 | KIF15 |  |  | 1213 | -0.610 | -0.5114 | Yes |
| 68 | BEX1 |  |  | 1216 | -0.615 | -0.5033 | Yes |
| 69 | CKS2 |  |  | 1226 | -0.641 | -0.5007 | Yes |
| 70 | NCAPH |  |  | 1228 | -0.643 | -0.4914 | Yes |
| 71 | ANLN |  |  | 1229 | -0.646 | -0.4812 | Yes |
| 72 | RAMP1 |  |  | 1230 | -0.648 | -0.4710 | Yes |
| 73 | FAM210B |  |  | 1233 | -0.652 | -0.4624 | Yes |
| 74 | CENPA |  |  | 1243 | -0.679 | -0.4592 | Yes |
| 75 | CDCA5 |  |  | 1244 | -0.685 | -0.4484 | Yes |
| 76 | SGO1 |  |  | 1246 | -0.691 | -0.4384 | Yes |
| 77 | APLP1 |  |  | 1249 | -0.700 | -0.4290 | Yes |
| 78 | AURKB |  |  | 1250 | -0.700 | -0.4180 | Yes |
| 79 | CCNA2 |  |  | 1255 | -0.716 | -0.4100 | Yes |
| 80 | UBE2T |  |  | 1256 | -0.719 | -0.3987 | Yes |
| 81 | CDCA2 |  |  | 1258 | -0.727 | -0.3881 | Yes |
| 82 | MMP2 |  |  | 1260 | -0.733 | -0.3774 | Yes |
| 83 | ARHGAP11A |  |  | 1262 | -0.735 | -0.3667 | Yes |
| 84 | HMGB2 |  |  | 1263 | -0.736 | -0.3551 | Yes |
| 85 | KIF23 |  |  | 1265 | -0.741 | -0.3443 | Yes |
| 86 | KIFC1 |  |  | 1267 | -0.748 | -0.3334 | Yes |
| 87 | TACC3 |  |  | 1268 | -0.751 | -0.3216 | Yes |
| 88 | ZMAT3 |  |  | 1269 | -0.759 | -0.3097 | Yes |
| 89 | GDF15 |  |  | 1280 | -0.828 | -0.3049 | Yes |
| 90 | PCLAF |  |  | 1281 | -0.828 | -0.2918 | Yes |
| 91 | ESCO2 |  |  | 1283 | -0.846 | -0.2794 | Yes |
| 92 | HJURP |  |  | 1290 | -0.897 | -0.2702 | Yes |
| 93 | SPC25 |  |  | 1294 | -0.932 | -0.2580 | Yes |
| 94 | RRM2 |  |  | 1295 | -0.933 | -0.2434 | Yes |
| 95 | KPNA2 |  |  | 1297 | -0.972 | -0.2289 | Yes |
| 96 | PIF1 |  |  | 1299 | -0.987 | -0.2142 | Yes |
| 97 | NCAPG |  |  | 1303 | -1.015 | -0.2007 | Yes |
| 98 | CDKN1A |  |  | 1306 | -1.046 | -0.1859 | Yes |
| 99 | CDK1 |  |  | 1311 | -1.433 | -0.1667 | Yes |
| 100 | UBE2C |  |  | 1312 | -1.466 | -0.1437 | Yes |
| 101 | TOP2A |  |  | 1313 | -1.528 | -0.1197 | Yes |
| 102 | SPARCL1 |  |  | 1314 | -1.537 | -0.0955 | Yes |
| 103 | NEAT1 |  |  | 1315 | -1.543 | -0.0713 | Yes |
| 104 | HIST1H1B |  |  | 1318 | -1.922 | -0.0427 | Yes |
| 105 | HIST1H4C |  |  | 1320 | -2.769 | 0.0000 | Yes |
Table: GSEA details [plain text format]

  

Fig 2: DALMO\_BARCODENUMBER\_UNPUB: Random ES distribution      
 Gene set null distribution of ES for **DALMO\_BARCODENUMBER\_UNPUB**
